# The natural course of newborns with transient congenital hypothyroidism

**DOI:** 10.1530/EC-24-0316

**Published:** 2024-11-21

**Authors:** Tal Almagor, Shlomo Almashanu, Ghadir Elias-Assad, Osnat Admoni, Hanna Ludar, Shira London, Shoshana Rath, Alina German, Naama Shwartz, Yardena Tenenbaum-Rakover

**Affiliations:** 1 Pediatric Endocrine Institute, Ha’Emek Medical Center, Afula, Israel; 2 The National Newborn Screening Program, Ministry of Health, Tel Hashomer, Ramat Gan, Israel; 3 Pediatric Endocrine Center, Clalit Health Services, Northern Region, Israel; 4 Pediatric Health Center, Clalit Health Services, Haifa, Israel; 5 Pediatric Endocrinology Service, Northern Health Center, Poria, Israel; 6 Department of Epidemiology, Carmel Medical Center, Haifa, Israel; 7 The Ruth & Bruce Rappaport Faculty of Medicine, Technion, Haifa, Israel; 8 Children's Endocrinology Consulting Center, Clalit Health Services, Afula, Israel

**Keywords:** congenital hypothyroidism, transient congenital hypothyroidism, permanent congenital hypothyroidism, newborn screening, thyroid function tests

## Abstract

**Objectives:**

The incidence of congenital hypothyroidism (CH) has increased worldwide over the last decades, mainly due to the lowering of screening thresholds, resulting in the increased identification of newborns with transient CH. Several studies have reported the prevalence and the predictive parameters of transient CH, but reports on the long-term outcome are rare. This study aimed to assess the long-term course of neonates with transient CH.

**Design:**

Neonates diagnosed with transient and permanent CH between the years 1998 and 2018 at the Pediatric Endocrine Institute of Ha’Emek Medical Center were enrolled in the study. Data were retrieved retrospectively from medical files.

**Results:**

A total of 76 newborns (45M, 59%) with transient CH and 53 (25M, 47%) with permanent CH were included in the study. The major causes of transient CH were prematurity (29%) and subclinical hypothyroidism (30%). During retrospective follow-ups of up to 23 years, reinitiation of levothyroxine therapy was not required, apart from four patients with underlying syndromic etiologies. Neurodevelopmental impairment occurred in 16% of children with transient CH compared with 29.4% in the permanent CH group.

**Conclusions:**

Transient CH is frequent among preterm infants but is generally limited to infancy. Subclinical hypothyroidism frequently presents as overt hypothyroidism at birth, but in most cases, the requirement for levothyroxine supplemental therapy is limited to the first years of life, suggesting that long-term follow-up of thyroid function tests may be unnecessary for non-syndromic children. The high rate of neurodevelopmental impairment in newborns with transient CH emphasizes the need for neurodevelopmental monitoring in these patients.

**Significance statement:**

A high rate of transient CH has been identified over the past decades following the lowering of TSH screening thresholds. The long-term outcome of transient CH has been evaluated in a few studies with inconclusive results. In the current study, we assessed the long-term outcomes of transient CH for up to 23 years. We found that 29% of cases were attributed to prematurity and 30% to subclinical hypothyroidism. No morphological anomalies were identified. Only syndromic patients (three with Down syndrome and one with Coffin-Lowry syndrome) required levothyroxine supplemental therapy at the time of the study, indicating that long-term thyroid function monitoring may be unnecessary. The high prevalence of neurodevelopmental impairment suggests the need for close neurodevelopmental monitoring in this population.

## Introduction

The incidence of congenital hypothyroidism (CH) has increased worldwide over the last decades (1, 2, 3, 4, 5, 6, 7, 8, 9, 10), mainly due to increased detection following the lowering of screening thresholds (1, 2, 3, 4, 7). In Israel, the prevalence of CH in the year 2022 was 1:1163 live births; however, the proportion of transient CH among them is unknown. Lowering of screening thyroid-stimulating hormone (TSH) cut-off resulted in a higher rate of transient CH diagnosis (1, 11, 12) since levels of TSH in neonates with transient CH are lower than in those with permanent CH (12, 13, 14, 15, 16, 17, 18, 19). Transient CH refers to CH that spontaneously resolves within the first few months or years of life (11). Currently, according to the 2020–2021 consensus guidelines of the European Society for Pediatric Endocrinology and the European Society for Endocrinology, the diagnosis of transient CH is established following the withdrawal of levothyroxine (LT4) therapy around 3 years of age (20). Between 5.3% and 52% of infants diagnosed with CH through newborn screening (NBS) programs were later determined to have transient CH (12, 13, 14, 15, 17, 18, 19, 21, 22), and among individuals with a gland *in situ*, the incidence was up to 73% (14, 17, 23, 24). Transient CH has been associated with lower screening TSH (12, 13, 14, 15, 16, 17, 18, 19, 21, 24) and higher FT4 levels at diagnosis (14, 15, 28), lower LT4 requirements (12, 13, 14, 15, 16, 18, 21, 22, 23, 24, 26, 27, 28, 29), lack of TSH elevation during treatment (18), low birth weight (21, 23, 25, 30), non-white ethnicity (30), male gender (21), prematurity (22, 23, 24, 25), neonatal intensive care unit (NICU) admission (30), maternal thyroid disease (18, 22), intake of antithyroid drugs (22), elevated serum thyroglobulin levels (21) and cardiac malformations (24). The precise etiology of transient CH is unknown in most cases, but some medical conditions have been reported to be associated with transient CH (3, 11), including iodine deficiency (11, 17) and excess (11), maternal transfer of TSH receptor-blocking (TRAb) autoantibodies (32, 33, 34, 35), and mutations in genes that are involved in thyroid hormone biosynthesis, including *DUOX2*, *DUOXA2* (36, 37, 38), and TSH receptor (*TSHR*) (39). Although several studies report on the predictive parameters and prevalence of transient CH, the long-term outcome of newborns with transient CH has been evaluated in few studies (40, 41, 42, 43). Intelligence quotient (IQ) measurements at the age of 7–8 years in infants with transient CH revealed lower IQ compared to controls (42). Longitudinal follow-up of newborns with mild congenital hyperthyrotropinemia revealed a high rate of thyroid morphology alterations, mild positive thyroid autoantibodies, and slightly persistent elevated TSH, suggesting that these patients exhibit minor congenital thyroid function abnormalities requiring long-term follow-up (43). In the current study, we evaluated retrospectively the long-term outcomes of 76 newborns with transient CH compared with 53 newborns with permanent CH and assessed the requirement for re-initiation of supplemental LT4 therapy for up to 23 years.

## Subjects and methods

### Subjects

Newborns who were diagnosed with transient CH between the years 1998 and 2018 at the Pediatric Endocrine Institute of Ha’Emek Medical Center were retrospectively enrolled. Newborns with TSH levels above 10 mIU/L in the first month of life (either on NBS or confirmatory laboratory TSH) were referred for endocrine assessment and included in the study. Transient CH was diagnosed when hypothyroidism resolved spontaneously within the first 3 years of life. Patients were diagnosed with permanent CH if they continued LT4 therapy subsequent to the LT4 withdrawal period between the ages of 2–3 years. Data were collected retrospectively from computerized medical files including the following parameters: sex, origin, consanguinity, maternal thyroid disease, LT4 therapy during pregnancy, thyroid disease in the extended family (any thyroid disease in the parents, grandparents, or siblings), high-risk pregnancy (maternal toxemia, gestational diabetes, twin pregnancy, abnormal findings on ultrasound screening, and *in vitro* fertilization-induced pregnancies), cesarean delivery, gestational age (GA), preterm newborns (GA < 37 weeks), birth weight, perinatal complications, NICU admissions (due to meconium aspiration, fetal respiratory distress, and other perinatal complications), and symptoms of hypothyroidism (mainly neonatal jaundice). Hormonal parameters included screening total T4 (TT4) and TSH, as well as laboratory TSH and free T4 (FT4), thyroglobulin, and thyroid autoantibodies (when available) at referral. LT4 therapy was withdrawn at the age of 2–3 years in all patients, and TSH and FT4 were measured. The occurrence of elevated TSH during follow-up, age, and dose of LT4 at the initiation of therapy, and last reported thyroid function tests (TFTs) in the medical files were collected. Imaging data included thyroid ultrasound and/or ^99m^TC scan when available. Neurodevelopmental impairment (including delayed psychomotor development, delayed language acquisition, attention deficit hyperactivity disorder (ADHD), and learning disorders) was based on assessments by either developmental pediatricians, pediatric neurologists, or reported therapeutic interventions in the Child Development Center. Patients with Down syndrome and Coffin–Lowry were excluded from the neurodevelopmental comparison between transient and permanent CH. A control group of 53 patients with permanent CH was selected at random from a cohort of 185 patients followed at our institute over the same period. The ethnicity of the control group was similar to that of the transient CH group. LT4 therapy was initiated immediately after laboratory confirmation of NBS identification of hypothyroidism. In some newborns, based on individual physician decisions, LT4 therapy was not given either because confirmatory laboratory TFTs, including TSH and FT4, were within the normal range, or due to the presence of elevated TSH with normal FT4 levels. In the latter, repeated TFTs were performed to avoid missing the development of overt hypothyroidism. LT4 was withdrawn between the ages of 2 and 3 years, and TFTs were assessed after 4 weeks without therapy to distinguish between permanent and transient CH according to the updated guidelines (20). If TFTs were within the normal range, LT4 supplementation was ceased. At the time of LT4 withdrawal, patients underwent a ^99m^Tc scan to investigate the presence, location, and size of the thyroid gland.

Laboratory TFTs were measured in infants born to mothers with thyroid diseases, preterm newborns, and those with Down syndrome in accordance with our neonatal department’s protocol at the time of the study (44). Subclinical hypothyroidism (SCH) (also known as compensated hypothyroidism or mild hypothyroidism) was defined as persistently elevated TSH levels in the presence of normal FT4 levels while off LT4 therapy.

### Neonatal screening

Blood samples were collected by heel puncture 48–72 h after birth. Between 1987 and 2006, the Israeli National Newborn Screening Laboratory performed the tests using Diagnostic Products Corp. (Los Angeles, CA, USA) radioimmunoassay TT4 and TSH kits. Since 2006, Perkin Elmer B065-112 AutoDELFIA neonatal TT4 and B032-312 AutoDELFIA neonatal TSH kits have been employed, both of which utilize time-resolved fluoroimmunoassays (PerkinElmer Life and Analytical Sciences, Wallac Oy, Turku, Finland). The Israeli NBS program measures TT4 levels, followed by a confirmatory TSH test when TT4 is below the 10^th^ percentile. TSH values above 20 mIU/L are considered indicative of primary CH, and results are immediately reported to the pediatric endocrinologist in charge of the geographical area. For newborns in NICU or with birth weight ≤2500 g or ≤36 weeks GA, TSH measurement is performed first, with confirmatory TT4 measurement performed when TSH is found to be above 23 mIU/L. National NBS results were retrieved retrospectively from computerized data for all newborns.

### Hormone analyses

TSH and FT4 were measured by a direct automated chemiluminescent immunoradiometric assay using the ADVIA Centaur immunoassay system (Bayer Corporation). TSH reference values provided by our laboratory were 0.4–4.2 mIU/L, and for FT4, 10–20 pmol/L. Normal references for the first week of life in our laboratory were 0.4–10 mIU/L for TSH and 10–26.8 pmol/L for FT4. Thyroglobulin, thyroglobulin antibodies (TGAb), and thyroid peroxidase (TPO) antibodies (TPOAb) were measured by a direct automated chemiluminescent immunoradiometric assay using an Immulite 2000 immunoassay system (Siemens). Thyroglobulin’s normal value is <55 ng/mL. TGAb and TPOAb levels above 35 U/mL were considered positive.

### Molecular analysis

When inherited thyroid disease was suspected based on a family history of CH, either a next-generation sequencing (NGS) panel or targeted gene sequencing of inherited thyroid diseases was performed. The NGS panel included genes related to thyroid diseases. Genetic analysis was performed only when available.

### Statistical analysis

The statistical analysis and data management were performed using SAS 9.4 software (SAS Institute Inc., Cary, NC, USA). Comparisons between the study groups were performed using the chi-square test (or Fisher’s exact test) for categorical variables and a* t*-test (or Wilcoxon rank-sum test) for continuous variables. *P* < 0.05 was considered significant.

The study was performed in line with the principles of the Declaration of Helsinki. Approval was granted by the Institutional Review Board of Ha’Emek Medical Center. Because there was no identification of subjects from whom data were retrieved, informed consent was waived.

## Results

Seventy-six newborns (45M, 59%) with transient CH and 53 (25M, 47%) with permanent CH were enrolled in the study. A comparison of demographic and clinical parameters between the groups is presented in Table 1. Neonates with transient CH had a higher rate of maternal thyroid disease (17% vs. 2%, *P* = 0.0053), maternal LT4 supplemental therapy during pregnancy (13% vs. none, *P* = 0.0098), high-risk pregnancy (39% vs. 18%, *P* = 0.0189), and prematurity (32% vs. 9.8%, *P* = 0.0083). A higher prevalence of NICU admissions and lower GA were found in patients with transient CH; however, the difference did not attain statistical significance. Hormonal results are presented in Table 2. Mean screening TSH was lower and TT4 higher in newborns with transient CH (38 mIU/L vs. 287, *P* < 0.001, 9.0 µg/dL vs. 5.1, *P* < 0.0001, respectively), and this difference was confirmed by laboratory FT4 and TSH levels (59.4 mIU/L vs. 297.5, *P* < 0.001; 14.2 nmol/L vs. 8.3, *P* < 0.001, respectively). Confirmatory laboratory TSH at diagnosis revealed TSH levels of less than 10 mIU/L in six newborns (CH was suspected in NBS results) and TSH above 10 mIU/L in 67 newborns. Among them, 49 had FT4 levels above 10 pmol/L, and 19 had FT4 less than 10 pmol/L. Mean thyroglobulin levels at referral were higher in the transient group (1044 ng/mL vs. 349, *P* = 0.0007). At LT4 withdrawal, as expected, mean FT4 levels were significantly lower in permanent CH (7.3 pmol/L vs. 16.2, *P* < 0.001), while their TSH was higher (245.4 mIU/L vs. 4.4, *P* < 0.001). A higher rate of elevated TSH during follow-up was observed in the permanent CH group (98% vs. 49%, *P* < 0.001). The last reported TFTs of patients with transient CH who were no longer treated with LT4 replacement revealed a mean FT4 of 15.7 nmol/L (range, 10.6–23.2) and mean TSH of 4.0 mIU/L (range, 0.5–10.6). Only four out of 53 newborns who underwent measurement of thyroid autoantibodies from the transient CH group were positive at referral, and no difference was found between the two groups. The clinical findings during referral and follow-up are presented in Table 3. LT4 treatment was initiated later in newborns with transient CH (32.5 days vs. 7.6, *P* < 0.0001), and they received a lower dose of LT4 at initiation (10.3 µg/kg/d vs. 12.3,* P* = 0.007). Of the newborns with transient CH, 20 (26%) were frequently monitored with TFTs without initiating LT4 therapy. The ^9^ ^9m^Tc scan demonstrated a normal thyroid gland in 29 patients and minor abnormal findings in five patients, including low uptake, higher uptake of one lobe, and a non-homogenous gland. The ^99m^Tc scan of patients with permanent CH revealed abnormal results in 24 patients (22 thyroid dysgenesis) and a normal-sized gland in a normal position in seven patients. Thyroid ultrasound demonstrated a normal thyroid gland in all patients with transient CH and an abnormal gland in 14 of 21 patients (62%) with permanent CH who underwent imaging. The timing of LT4 withdrawal did not differ between the groups (2.5 years vs. 2.7). As expected, the mean follow-up duration was longer in permanent CH (mean, 6.7 years vs. 9.3, *P* = 0.002), lasting up to 23 years. Patients with permanent CH had a higher rate of neurodevelopmental impairment; however, the difference did not reach statistical significance (16.7% vs. 29.4%, *P* = 0.0766). Among the 11 subjects with neurodevelopmental impairment in the transient CH group, four were born prematurely. Additional anomalies and other acquired diseases were observed in the two groups at the same rate. At the time of the study, four patients (5%) with transient CH had reinitiated LT4 therapy (three with Down syndrome and one with Coffin–Lowry syndrome). In these four patients, LT4 was ceased at the age of 2–3 years and later reinitiated by their family physician or the pediatric endocrinologist during the follow-up period. One patient with a heterozygous probably pathogenic variant of the *DOUX2* gene had persistent elevated TSH during follow-up, and LT4 therapy was reinitiated. At the time of the study, he was off LT4 therapy. All patients in the permanent CH group continued LT4 therapy. All newborns with permanent CH were detected by the NBS program apart from one infant in whom TFTs were measured because of prematurity. In the transient CH group, only 47 patients (62%) were detected by the NBS program. CH etiologies for patients from the two groups are summarized in Table 4. Thirty-one patients (58%) from the permanent CH group had thyroid dysgenesis, 22 (42%) had eutopic thyroid glands with dyshormonogenesis identified in eight patients, SCH in two, and unknown etiology in 12 patients. In the transient CH group, etiologies included prematurity (22), SCH (23), Down syndrome (7), maternal thyroid disease (3), screening false positive (3), *DUOX2* variants (2), familial transient CH (1), maternal Betadine use (1), and unknown etiology (22).

**Table 1:** Comparison of clinical characteristics between transient and permanent congenital hypothyroidism.

Mean ± s.d. (range) No. (%)	Transient CH	Permanent CH	*P*
Number of patients.	76	53	
Sex	31F/45M	28F/25M	0.1768
Ethnic origin	36J/36AM/2AC/2D	23J/27AM/2AC/1D	0.9348
Consanguinity^a^	10 (14%)	13 (26%)	0.1166
Maternal thyroid diseases	12 (17%)^b^	1 (2%)	0.0053
Maternal LT4 therapy	9 (13%)	0	0.0098
Thyroid disease in the extended family	23 (33%)	20 (40%)	0.4211
High-risk pregnancy	27 (39%)^c^	8 (18%)	0.0189
Cesarean delivery	21 (30%)	11 (25%)	0.5318
Prematurity	22 (32%)	4 (9.8%)	0.0083
GA	37.9 ± 2.9 (28–42)	38.7 ± 3.0 (25–42)	0.0677
Birth weight	2874 ± 784 (645–4720)	3001 ± 703 (790–4600)	0.3760
Perinatal complications	30 (43.5%)^d^	12 (29.3%)	0.1380
NICU	26 (38%)	9 (20%)	0.0559
Symptoms at presentation	19 (31%)^e^	17 (46%)^f^	0.1257

^a^The prevalence of consanguinity among the Arab population in the North region of Israel in year 2010 was 31%.

^b^Hypothyroidism (9) and SCH (3).

^c^IVF (4), twins (8), ultrasound scan revealed fetal anomalies (7), and complicated pregnancies (14).

^d^Small for GA, meconial aspiration, respiratory distress syndrome, hypoglycemia, and polycythemia.

^e^Neonatal jaundice (14), feeding problems (3), and hypotonia (3).

^f^Neonatal jaundice (11), macroglossia (3), hypotonia (3), and umbilical hernia (2).

AM, Arab-Muslim; AC, Arab-Christian; D, Druze; CH, congenital hypothyroidism; F, females; GA, gestational age; IVF, *In vitro* fertilization; J, Jewish; M, males; NICU, newborn intensive care unit; SCH, subclinical hypothyroidism.

**Table 2:** Comparison of thyroid function test between transient and permanent congenital hypothyroidism at presentation and during follow-up.

Hormonal results	Transient CH No. (%) or mean ± s.d. (range)	Permanent CH No. (%) or mean ± s.d. (range)	*P*
Screening TT4 (µg/dL)	9.3 ± 4.2 (1.1–21.0)	5.1 ± 2.9 (1.3–10.3)	<0.0001
Screening TSH (mIU/L)	38.0 ± 41.8 (5–244)	287.1 ± 195.5 (7-619)	<0.001
Confirmatory FT4 (pmol/L)	14.2 ± 6.2 (3–39.7)	8.3 ± 3.9 (0.4–16.0)	<0.001
Confirmatory TSH (mIU/L)	59.4 ± 62.6 (4.0–297.0)	297.5 ± 236.9 (45.0–931.0)	<0.001
Thyroglubin (ng/mL)	1044.4 ± 2001.5 (32.0–13106.0)	348.7 ± 634.0 (14.8–2656.0)	0.0007
Age at confirmatory TFTs (days)	14.3 ± 10.6 (2.0–49.0)	7.8 ± 4.7 (4.0–30.0)	<0.001
Positive TPOAb	3 (5.9%)	3 (11.1%)	0.4116
Positive TGAb	2 (4.1%)	0	0.5409
FT4 at LT4 cessation (pmol/L)	16.2 (12–23)	7.3 ± 5.1 (1.3–17.4)	<0.001
TSH at LT4 cessation (mIU/L)	4.4 ± 2.7 (0.7–13.0)	245.4 ± 179.8 (8.5–651.4)	<0.001
Patient follow-up with high TSH	35 (49.3%)	50 (98%)	<0.001
Age at Last TFTs (year)	5.9 ± 4.9 (0.1–23.0)	9.4 ± 4.9 (3.0–23.0)	<0.001
Last FT4 (pmol/L)	15.7 ± 2.3 (10.6–23.3)	18.8 ± 4.4 (3.5–25.6)	<0.001
Last TSH (mIU/L)	4.0 ± 2.0 (0.5–10.6)	26.2 ± 77.6 (0.2–454.0)	0.6313

CH, congenital hypothyroidism; FT4, free T4; LT4, levothyroxine; TGAb, thyroglobulin antibodies; TPOAb, thyroid peroxidase antibodies; TFTs, thyroid function tests; TT4, total T4.

**Table 3:** Comparison of clinical characteristics during follow-up between children with transient and permanent congenital hypothyroidism.

Mean ± s.d. (range) No. (%)	Transient CH	Permanent CH	*P*
Age at LT4 initiation (days)	32.5 ± 88.2 (1.1–580.0)^a^	7.6 ± 5.2 (4.0–30.0)	<0.0001
No therapy	20 (26%)	0	
LT4 dose at initiation (µg/kg/d)	10.3 (1.4–24)	12.3 ± 2.9 (7.5–18.0)	0.0070
^99^^m^Tc scan	34 (44.7%) (abnormal 5/normal 29)	31 (58%) (abnormal 24/normal 7)	0.0002
	Non-homogenous (1) Low uptake (2) Right lobe uptake more than the left lobe (2)	Agenesis (5) Ectopic (15) Hypoplastic (2) Low uptake (2)	
Ultrasound imaging	19 (25%) Abnormal 0/Normal 19	21 (39.6%) Abnormal 14/Normal 7	0.0266
Age at LT4 cessation (years)	2.5 ± 1.7 (0.3–11.3)	2.7 ± 1.0 (2.0–6.0)	0.1480
Follow-up duration (years)	6.7 ± 5.2 (0–23.0)	9.3 ± 5.0 (3.0–23.0)	0.0020
Neurodevelopment impairment	11 (16.7%)^b^	15 (29.4%)	0.0766
Other diseases	22 (32.8%)^c^	11 (21.6%)^d^	0.1767
LT4 replacement therapy at the time of study	4 (5%)^e^	All	
Reasons for performing TFTs	Newborn screening 47 (62%) Maternal thyroid diseases 7 (9.2%) Symptoms of hypothyroidism 11 (14.5%) Down syndrome 5 (6.6%) Siblings with CH 2 (2.6%) Unknown 3 (4%)	Newborn screening 52 (98.1%) Prematurity 1(1.9%)	<0.0001

^a^Screening TFTs revealed TSH of 20 mIU/L and TT4 of 7.9 µg/dL. Laboratory TFTs at the age of 25 days revealed TSH of 10.6 mIU/L and FT4 of 14.2 nmol/L. He was initiated on LT4 therapy only at the age of 580 days and stopped therapy at the age of 2.75 years. At the age of 10.3 years without LT4 therapy, TSH was still elevated at 8.5 and FT4 14.4 nmol/L. Molecular analysis identified a heterozygous mutation of the TSH receptor, p.L653V, that was identified previously in his extended family.

^b^Delayed psychomotor development, delayed language acquisition, ADHD, and learning disorders.

^c^Vitiligo, cardiac anomalies, Celiac disease, Down syndrome (7), short stature, and other syndromes.

^d^Nephrotic syndrome, diabetes mellitus type 1, familial hypocalciuric hypercalcemia, celiac disease, coloboma, hearing loss, and autism spectrum disorder autism.

^e^Down syndrome (3) and Coffin–Lowry syndrome (1).

ADHD, attention deficit disorder; CH, congenital hypothyroidism; ND, not done; TFTs, thyroid function tests.

**Table 4:** Summary of estimated etiologies for transient CH and the etiologies of persistent CH.

Transient CH		Permanent CH		
Estimated cause for CH	No. (%)	Etiology of CH		No. (%)
Prematurity^a^	22 (29)	Thyroid dysgenesis 31 (58%)	Ectopic	16 (30)
SCH	23 (30)		Agenesis	6 (11.3)
Down syndrome	7 (9.2)		Hypoplastic	7 (13)
UN	22 (29)		Hemi-thyroid	2 (3.8)
Maternal thyroid ab’s	3 (3.9)	Eutopic thyroid 22 (42%)	Dyshormonogenesis^c^	8 (15%)
Delayed diagnosis of CH	2 (2.6)		TSHR	2 (3.8)
Familial transient CH	1 (1.3)		Unknown	12 (22.6)
Screening FP	3 (3.9)			
*DUOX2* and *DOUXA2* variants^b^	2 (2.6)			
Maternal betadine use	1 (1.3)			

^a^Eight patients were premature and had SCH as well.

^b^One patient had two heterozygous variants in the *DOUXA2* gene inherited from her unaffected father (splice mutation c.205+2T>C and missense mutation c.463C>G, p.L155V). Screening TFT revealed TSH of 33.4 mIU/L and TT4 of 8.9 µg/dL. Laboratory TFT at the age of 9 days revealed TSH of 38.3 mIU/L and FT4 of 15.0 nmol/L. At the age of 16.7 years without LT4 therapy, TSH was 3.3 and FT4 was 16.7. The second patient had a heterozygous p.P303R variant in the *DOUX2* gene that was inherited from his mother who had mild hyperthyrotropinemia. Screening TFT revealed TSH of 13.8 mIU/L and TT4 of 15.5 µg/dL. Laboratory TFT at the age of 49 days revealed TSH of 38.8 mIU/L and FT4 of 15.5 nmol/L. At the age of 17 years without LT4 therapy, TSH was elevated at 9.62 and FT4 at 14.75. The variants that were identified are likely pathogenic.

^c^Four patients with *TPO* mutation and one with *TG* mutation.

CH, congenital hypothyroidism; FP, false positive; SCH, sub-clinical hypothyroidism; TPO, thyroid peroxidase; TG, thyroglobulin; TSHR, TSH resistance; UN, unknown.

## Discussion

Our long-term follow-up of newborns with transient CH indicates that the reintroduction of LT4 occurs only in those with a syndromic etiology. Transient CH was associated with SCH in 30% of cases and prematurity in 29% of subjects. Our results accord with previous studies concerning predictive parameters for transient CH, which include lower TSH (12, 13, 14, 15, 16, 17, 18, 19, 21, 24, 27, 28) and higher FT4 levels at diagnosis (14, 15, 28), lower LT4 requirements (12, 13, 14, 15, 16, 18, 21, 22, 23, 24, 26, 28, 29), and prematurity (22, 23, 24, 25). Prematurity was reported in 32% of those with transient CH and appears to be a major contributor to the increased prevalence of CH in recent years. Prematurity is associated with a blunted TSH surge and a delay in the timing and magnitude of serum T4 and total T3 levels, correlating with GA (25). In most preterm babies, serum TT4 levels gradually rise and match those seen in term babies by 4–8 weeks of age. Due to this physiologic mechanism, the Israeli NBS program uses first-line TSH measurement instead of TT4 in preterm newborns. Despite this, elevated TSH with either low or normal FT4 concentrations was identified at a high rate in premature infants in our cohort, necessitating LT4 replacement therapy. Another important etiology of transient CH is iodine deficiency (11, 31) or excess. Iodine use is avoided in our obstetrics and neonates departments, minimizing the incidence of thyroid function complications; however, one preterm infant was born in another hospital and was diagnosed with transient CH secondary to maternal iodine exposure during an emergency cesarean section. Maternal transfer of TRAb is a rare cause of transient CH, estimated to account for 2% of infants with CH (35). In our cohort, three newborns (4%) had transient CH associated with maternal Hashimoto’s thyroiditis. In these babies, thyroid autoantibodies (TPOAb and TGAb) were positive at referral; however, TRAb was not measured, being available in only a few specialized laboratories. Down syndrome was reported in seven patients, all of whom had non-autoimmune SCH. While treatment ceased in all of them between the ages of 2–3 years, three required reinitiation of therapy at a later age. A high rate of thyroid disease, including permanent and transient CH, SCH, and autoimmune thyroid disease, has been reported in Down syndrome (45). The seven patients with Down syndrome had persistently elevated TSH levels, normal FT4 values, and no anti-thyroid antibodies, consistent with the diagnosis of non-autoimmune SCH. Indeed, guidelines recommend TSH measurement at 1 month of age in newborns with Down syndrome due to a CH incidence 14–21 times greater than the normal population (20).

Twenty-three (30%) newborns had mildly elevated TSH after LT4 withdrawal but did not require reinitiation of therapy, consistent with non-autoimmune SCH. This condition may result from loss-of-function mutations of the* TSHR* gene or post-receptor defects. Indeed, one patient with persistently elevated TSH was found to have a heterozygous loss-of-function mutation in the *TSHR* gene, previously reported in an extended consanguineous family in our region (46). Two patients had novel variants in the *DUOX2* and *DUOXA2* genes possibly deleterious (for details, see Table 4). Biallelic and monoallelic mutations in *DUOX2* and *DUOXA2* have been reported in patients with either transient or permanent CH (37, 38). Since *DUOX2* variants are found at high frequencies among healthy populations, it has been postulated that *DUOX2* and *DUOXA2* mutations may predispose to hypothyroidism in the setting of genetic or environmental modulators but are not directly causative (37, 38). One patient with the *DUOX2* variant had persistently elevated TSH and was therefore recommenced on LT4 therapy during follow-up. He did not experience any clinical benefit from therapy and therefore ceased treatment sometime later. At the time of the study, aged 17 years, he was off LT4 therapy.

It has been suggested that earlier treatment re-evaluation in neonates with a gland *in situ* requiring LT4 doses of less than 3 µg/kg per day at the age of 6 months may be appropriate (20, 21, 47). In our cohort, treatment was stopped at 2–3 years of age (mean 2.5 years) in accordance with current guidelines (20). Interestingly, we have shown that 16.7% of newborns with transient CH had neurodevelopmental impairment. These findings may be explained partially by the high prematurity rate and other underlying conditions associated with developmental delay; however, late initiation of LT4 therapy cannot be excluded as an additional contributor. Previous reports have shown delayed expressive language acquisition (41) and lower intelligence quotients in children aged 7–8 years diagnosed with transient CH in infancy (42). The high rate of neurodevelopmental impairment in the permanent CH group (29.4%) may be partially explained by the fact that our cohort included newborns who were diagnosed with CH over 23 years. Those born earlier were often diagnosed after 10 days, which is referred to as late diagnosis according to the 2020–2021 consensus guidelines (20).

This study is the first to review, although retrospectively, the outcome of newborns with transient CH for up to 23 years. Only syndromic patients required LT4 therapy at the time of the study, indicating that most patients with transient CH have temporary hypothyroidism in infancy without recurrence over time.

In a previous study by Köhler *et. al.* with a follow-up of up to 14 years, the authors concluded that ‘longitudinal surveys of TFTs should be avoided’ (40). In contrast, Leonardi *et al.*, found that newborns with mild hyperthyrotropinemia are at a higher risk for thyroid morphology alterations and have a high rate of persistent SCH, necessitating prolonged monitoring (43). We did not find any morphological abnormalities on ultrasound in our cohort, with only mildly abnormal findings on ^99m^Tc scans identified in five patients.

The strength of this study is the long-term follow-up duration, enabling us to observe the natural history of newborns with transient CH. The limitations of this study include its retrospective nature, with data collected over 23 years from medical records. In addition, molecular analysis was performed only on a few patients, and thyroid autoantibodies were not measured in all participants. Neurodevelopmental assessment in the study relied on evaluations provided by developmental pediatricians or pediatric neurologists. Not all children underwent uniform, validated psychological or neurological assessments.

In conclusion, prematurity and SCH were the major causes of transient CH in this study. Persistent TSH elevation is common in newborns with transient CH; however, overt hypothyroidism occurs only in infancy, obviating the need for long-term monitoring of thyroid function in most cases. The high rate of neurodevelopmental impairment identified in this cohort emphasizes the necessity of close neurodevelopmental monitoring in transient CH patients.

## Declaration of interest

The authors declare that there is no conflict of interest that could be perceived as prejudicing the impartiality of the research reported.

## Funding

This work did not receive any specific grant from any funding agency in the public, commercial, or not-for-profit sector.

## Data availability

Further data are available from the corresponding author upon reasonable request.

## Author contribution statement

Y-TR designed the study, analyzed and interpreted the data, and wrote the first draft. TA collected the data and reviewed and revised the manuscript. SA performed the NBS. G-EA, OA, SL, AG, SR, and HL followed the patients and reviewed and revised the manuscript. NS performed the statistical analysis. All authors approved the final manuscript and agreed to be accountable for the content of the work.
